# Direct Imaging of Lipid Metabolic Changes in *Drosophila* Ovary During Aging Using DO-SRS Microscopy

**DOI:** 10.3389/fragi.2021.819903

**Published:** 2022-02-03

**Authors:** Yajuan Li, Pegah Bagheri, Phyllis Chang, Audrey Zeng, Jie Hao, Anthony Fung, Jane Y. Wu, Lingyan Shi

**Affiliations:** ^1^ The Department of Bioengineering, University of California San Diego, La Jolla, CA, United States; ^2^ Department of Neurology, Northwestern University, Chicago, IL, United States

**Keywords:** stimulated Raman scattering, DO-SRS, lipid metabolism, aging, ovary, *Drosophila*, heavy water, metabolic dynamics

## Abstract

Emerging studies have shown that lipids and proteins play versatile roles in various aspects of aging. High-resolution *in situ* optical imaging provides a powerful approach to study the metabolic dynamics of lipids and proteins during aging. Here, we integrated D_2_O probing and stimulated Raman scattering (DO-SRS) microscopy to directly visualize metabolic changes in aging *Drosophila* ovary. The subcellular spatial distribution of *de novo* protein synthesis and lipogenesis in ovary was quantitatively imaged and examined. Our Raman spectra showed that early stages follicles were protein-enriched whereas mature eggs were lipid-enriched. DO-SRS imaging showed a higher protein synthesis in the earlier developing stages and an increased lipid turned over at the late stage. Aged (35 days) flies exhibited a dramatic decrease in metabolic turnover activities of both proteins and lipids, particularly, in the germ stem cell niche of germarium. We found an accumulation of unsaturated lipids in the nurse cells and oocytes in old flies, suggesting that unsaturated lipids may play an important role in the processes of oocyte maturation. We further detected changes in mitochondrial morphology and accumulation of Cytochrome c during aging. To our knowledge, this is the first study that directly visualizes spatiotemporal changes in lipid and protein metabolism in *Drosophila* ovary during development and aging processes. Our study not only demonstrates the application of a new imaging platform in visualizing metabolic dynamics of lipids and proteins *in situ* but also unravels how the metabolic activity and lipid distribution change in *Drosophila* ovary during aging.

## Introduction

Accumulating evidence indicates that the quantitative and qualitative changes in stem cells with time contribute to aging and age-related diseases (Katajisto et al., 2015; Hinnant et al., 2020; Na and Kim, 2020). Genetic and epigenetic pathways involved in stem cell maintenance, division, and differentiation have been identified to be evolutionarily conserved, including the hedgehog, wingless, JAK/STAT, insulin, and TGF-β (Hsu et al., 2008; Kosan and Godmann, 2016; Jeong et al., 2019). *Drosophila*, especially its ovary, provides a sophisticated model system to study stem cell aging (Knoblich, 2008; Katajisto et al., 2015; Hinnant et al., 2020; Tiwari and Mandal, 2021). There are two types of stem cells inside *Drosophila* ovary: the germ-line stem cells that generate oocytes and their supporting nurse cells, and the somatic stem cells that give rise to the surrounding follicular epithelium (Knoblich, 2008; Garcez et al., 2020; Hinnant et al., 2020; Doherty et al., 2021; Tiwari and Mandal, 2021). Similar to mammals, ovarian aging in *Drosophila* results in the decline of reproductive function that is characterized by reduced quality and quantity of eggs (Moghadam et al., 2021).

Among different mechanisms of ovarian aging, metabolic changes have attracted much attention since metabolism of ovary impacts the quality of oocyte and the development of embryo. Dysregulation of protein homeostasis during aging has been well understood as a causal factor of low-quality germ cells (Duncan and Gerton, 2018). For example, the oocytes generated from elder individuals have shown increased fibrillarin expression in humans (Yang et al., 2019; Wang and Na, 2021). Association of lipid metabolism in ovary development and aging has also been increasingly recognized. However, changes in metabolic activity and specific chemical molecule that influence the function of ovary during aging have not been clearly deciphered yet. Understanding this dynamics of metabolism during aging is important and will provide insights into molecular mechanisms underlying premature ovarian failure and infertility diseases (Zhang et al., 2019a; Baddela et al., 2020).

Common analytical and imaging techniques have limitations. For example, gas chromatography (GC) needs long-time sample preparation, mass spectrometry (MS)-based techniques or matrix-assisted laser desorption/ionization (MALDI)-MS imaging are destructive to live tissues, nuclear magnetic resonance (NMR) spectroscopy has relatively low spatial resolution, and fluorescence microscopy requires bulky fluorescent dyes that may disturb the activities of native molecules.

As a nondestructive technique, Raman spectroscopy has been widely used since the past decades, which is based on the inelastic scattering of light by vibrating molecules (Raman scattering). Different chemical bonds vibrate at different frequencies, giving distinct fingerprints of molecules in the Raman spectrum. Thus, each peak in a Raman spectrum corresponds to certain chemical bonds’ vibrational modes. Furthermore, the intensity of Raman scattering signal has a linear relationship to the concentration of a molecule’s chemical bonds, which allows for quantitative imaging. One drawback of spontaneous Raman is its weak signal intensity that greatly limits imaging speed for visualizing metabolic dynamics in living organisms. Stimulated Raman scattering (SRS) and heavy water (D_2_O) probed-SRS (DO-SRS) microscopy have emerged as new imaging techniques in the past decade with much faster imaging speed (∼1,000 times faster than spontaneous Raman scattering). SRS uses two laser sources to coherently excite the vibration of molecules. When the energy difference between these two laser beams matches the vibrational frequency of a molecule of interest in the tissues, the transition rate can be dramatically enhanced up to 10^8^-fold. With this level of sensitivity, either of these two beams can be used as the source for video rate imaging. SRS imaging is advantageous in its multiplex imaging capability, high subcellular resolution and chemical specificity, and noninvasiveness (Ghita et al., 2012; Kong et al., 2013; Ember et al., 2017). Heavy water (D2O) has been applied as a Raman probe to identify and sort metabolic activities of microbial cells by tracking the incorporation of D_2_O-derived deuterium labeled macromolecules into bacteria (Berry et al., 2015). We recently established a new imaging platform that combined D_2_O probing with SRS microscopy for *in situ* visualization of metabolic dynamics at subcellular scale in small animals (Shi et al., 2018; Bagheri et al., 2021; Li et al., 2021). As an isotopologue of water, D_2_O can diffuse freely into living cells and tissues, where the deuterium (D) from D_2_O will be incorporated with carbon (C) atoms to form C-D bonds in newly synthesized biomolecules such as lipids and protein through metabolism. The C-D bonds produce distinct peaks in the “cell-silent” region of Raman spectra (wavelength 1,800–2,800 cm^−1^), and by investigating the C-D vibrational spectra, we identified C-D bond-containing molecules. Furthermore, applying DO-SRS microscopy imaging we directly visualized the metabolic dynamics of proteins, lipids, and DNA in living cells and animals including *C. elegans*, zebrafish, *Drosophila*, and mouse. In this study, we first integrated D_2_O probing with Raman spectroscopy to identify C-D spectra of lipid and protein in aging *Drosophila* ovary, and then applied DO-SRS microscopy to image and track metabolic dynamics of *Drosophila* ovarian follicles *in situ* during development. We quantitatively monitored changes in lipid and protein biosyntheses. Moreover, by comparing the ratiometric images of different lipid subtypes in young and aged flies, we demonstrated the lipid profile changes in ovary during the aging process.

## Results

### Label-free Raman Imaging of *Drosophila* Ovariole Development

**FIGURE 1:**
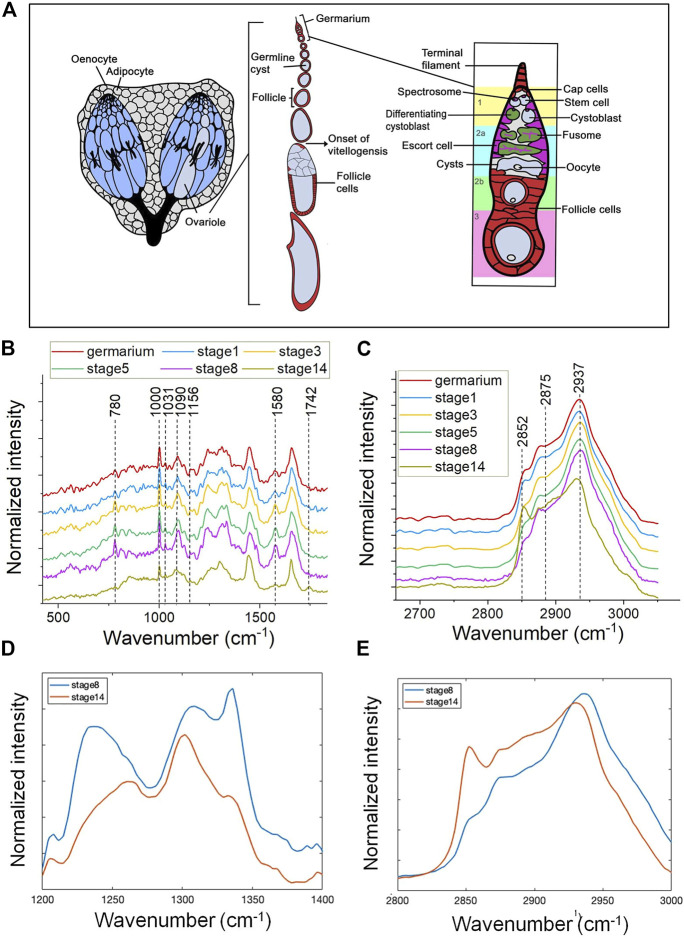
Spontaneous Raman spectra of *Drosophila* ovaries revealing molecular changes during oogensis. **(A)** A diagrammatic illustration of the structure of adult ovary. The *Drosophila* ovary consists of two lobes that are connected by a common oviduct. Each lobe is composited of approximately 14–16 ovarioles. Each ovariole harbors an anterior localized germarium and following older follicles that develop through 14 distinct stages. Within the germarium, germline stem cells (GSCs) are surrounded by cap cells and escort cells, two major cellular components of the somatic niche. One GSC generates two daughter cells through unsymmetric mitosis, one is GSC and the other one is cystoblast. Each cystoblast divides four additional times to form 16-cell germline cysts consisted of nurse cells (nc) and an oocyte (oo). Follicle stem cells (FSC) divide to form prefollicle cells, which surround the 16-cell germline cyst, and isolate them from the germarium. Prefollicle cells give rise to different kinds of specialized follicle cells (fc) that localized in an epithelial monolayer surrounding each cyst. The mature oocyte is localized at the posterior end of each ovariole. The fingerprint region **(B)** and CH stretching region **(C)** of mean Raman spectra (*n* = 10) of follicles at different developmental stages in 5-day female flies. **(B)** Compared with the mature oocyte, germarium and the developing follicles show a relatively high intensity of Raman peaks at 780 cm^−1^, 1,000 cm^−1^, and 1,580 cm^−1^ and these peaks show a gradually higher intensity alongside the follicle maturation. There is a shape remodeling of amide III in the last stage of oogenesis (stage 14), which is different from the early stages. **(C)** A dramatically high intensity at 2,852 cm^−1^ is shown in stage 14 oocytes and the peak position of stage 14 oocytes at 2,937 cm^−1^ is relatively left-shifted. **(D)** The enlarged amide III region and **(E)** CH stretching region comparing the Raman spectral shape from stage 8 (blue) and stage 14 (red) follicle. Early developing follicles (stage 8) showed more protein enriched, and mature eggs (stage 14) showed more likely lipid dominated.

The *Drosophila* ovary consists of two lobes that are connected by a common oviduct. Each lobe is composed of approximately 14–16 ovarioles where the ovariole harbors an anterior localized germarium, following older follicles. Within the germarium, germline stem cells (GSCs) are surrounded by the somatic cells. One GSC generates two daughter cells, a GSC and cystoblast, through unsymmetric mitosis. Each cystoblast divides to form nurse cells (nc) and an oocyte (oo). Follicle stem cells (FSC) divide and differentiate to different kinds of specialized follicle cells (fc) that support germ cells. The mature oocyte is localized at the posterior end of each ovariole (Figure 1A), and *Drosophila* oogenesis is fueled by the activity of germline stem cells (Hinnant et al., 2020).

We measured the Raman spectra from fixed ovarioles of 5-day-old females at 6 different stages: germarium, the developing follicle stage 1, stage 3, stage 5, stage 8, and the mature oocyte at stage 14 (Figure 1A) to determine biomolecular changes in distribution and quantity during development.

To have a more comprehensive picture Raman spectra were measured in the fingerprint region (400–1800 cm^−1^) and the CH stretching region (2,800–3,050 cm^−1^), respectively (Figures 1B,C). Assignment of prominent peaks is summarized in Table 1. In particular, we observed prominent changes at peaks 780 cm^−1^, 1,000 cm^−1^, and 1,580 cm^−1^, which correspond to the vibrational modes of C-O backbone in nucleic acids, the ring breathing mode of phenylalanine (Puppels et al., 1990), and Cytochrome c (Draga et al., 2010; Kumamoto et al., 2018; Hniopek et al., 2021), respectively.

**TABLE 1:** The chemical bond assignment of Raman peaks of *Drosophila* ovary.

	—
716–720 cm-1	Phosphatidylcholine
753 cm-1	Tryptophan
780–784 cm-1	DNA
818 cm-1	RNA/DNA backbone
850–856 cm-1	Glycogen or tyrosine
1,000 cm-1	Phenylalanine
1,031 cm-1	C-H in plane bending of Phenylalanine
1,087–1,090 cm-1	DNA backbone
1,125 cm-1	C-N or C-O carbohydrate
1,152–1,160 and 1,520 cm-1	Carotenoid
1,200–1,300 cm-1	Amide III: C-N stretching and N-H bending
1,260 cm-1 in Amide III	β-sheets and random coils
1,300 in Amide III	Α-helices
1,338 cm-1	Tryptophan
1,402 cm-1 (1,410 cm-1)	COO−symmetric stretch of aspartic and glutamic acid residues, indicates that most acid side chains are deprotonated
1,430 cm-1	CH2 scissoring
1,443 cm-1	CH2, CH3 deformation of lipids and triglycerides
1,453 cm-1	C-H bending of lipids
1,580 cm-1	Cytochrome c
1,580–1700 cm-1	Amide: stretching vibration of C=O
1,655	v (C=C) cis double bond stretching mode
1,676	Aβ
1740 cm-1	Stretching vibration from C=O in lipid, A shift to higher wavenumbers for longer chain length fatty acids
2,852 cm-1	CH2 symmetric stretching (lipids)
2,875 cm-1	CH2 asymmetric stretching (lipids, proteins)
2,920–2,937 cm-1	CH3 symmetric stretching (lipids)
2,959 cm-1	CH3 asymmetric stretching (lipids, proteins)
3,005 cm-1	Olefinic = CH stretching (unsaturated lipids, cholesterol esters)

Compared with the mature oocyte, germarium and developing follicles showed relatively high intensities at 780 cm^−1^, 1,000 cm^−1^, and 1,580 cm^−1^ (Figure 1B). This could be due to a higher content of nucleic acids, proteins, or Cytochrome c. In addition, the Raman signals observed at these three positions showed a gradually increasing intensity along with the maturation of the follicle. Furthermore, we found the intensity of these Raman signals peaked at stage 8 follicles and then reduced in mature eggs. Similarly, accompanied by follicle maturation, the intensity of amide III broad band increased (from germarium to the developing follicle stage 1, stage 3, stage 5, stage 8), but there was a shape remodeling of amide III in the last stage of oogenesis (stage 14), which is a classic lipid-enriched shape (Figures 1B,D). Consistently, the Raman bands present in the high wavenumber show a dramatically high intensity at 2,852 cm^−1^ in stage 14 oocytes, which was assigned to symmetric vibrations of the CH_2_ groups present in lipids (Figures 1C,E). The lipid accumulation of stage 14 detected by Raman spectra is consistent with the Nile red staining results presented by the previous studies (Parra-Peralbo and Culi, 2011; Sieber and Spradling, 2015).

The peak at 2,937 cm^−1^ was mainly due to the CH stretching mode of CH_3_ groups from proteins. It has a similar profile of proteins and lipids for both germarium and the developing follicles (stage 1, stage 3, stage 5, stage 8). The protein peak position of the mature oocytes was left-shifted as depicted in Figures 1C,E. The shift of the peak was possibly due to the large amount of yolk proteins interacting with lipids in a specific way, or the proteins being modified by lipids at this mature stage. However, further investigation is necessary to clarify this.

### DO-SRS Imaging of Metabolic Activity of *Drosophila* Ovariole During Aging

The female flies continuously produce hundreds of eggs throughout their lifespan (Rogina et al., 2007). The continued oocyte production requires consecutive division of the stem cells. The egg production is dramatically decreased during female aging, indicating a significant compromise in the rate of stem cell division (Ren et al., 2017; Dipali et al., 2019; Armstrong, 2020). Both autonomous signals from a defect in the stem cells and the nonautonomous signals sent to the germ stem cells from the surrounding niche can contribute to replicative senescence (Narbonne, 2018; Sênos Demarco and Jones, 2019). It has been suggested that the nutrient status of *Drosophila* determines the ovarian function, as enormous energy is required to fuel the oogenesis (Pritchett et al., 2009; Jeong et al., 2019; Armstrong, 2020). The aged flies have impaired physical activity and reduced digestive function, which suggests that malnutrition may contribute to ovarian senescence during aging. In addition, some studies have shown the morphology and dynamics of mitochondria changing during ovarian aging (May-Panloup et al., 2016; Garcez et al., 2020). However, the direct evidence of subcellular metabolic activity changes during ovarian aging has not been revealed.

**FIGURE 2:**
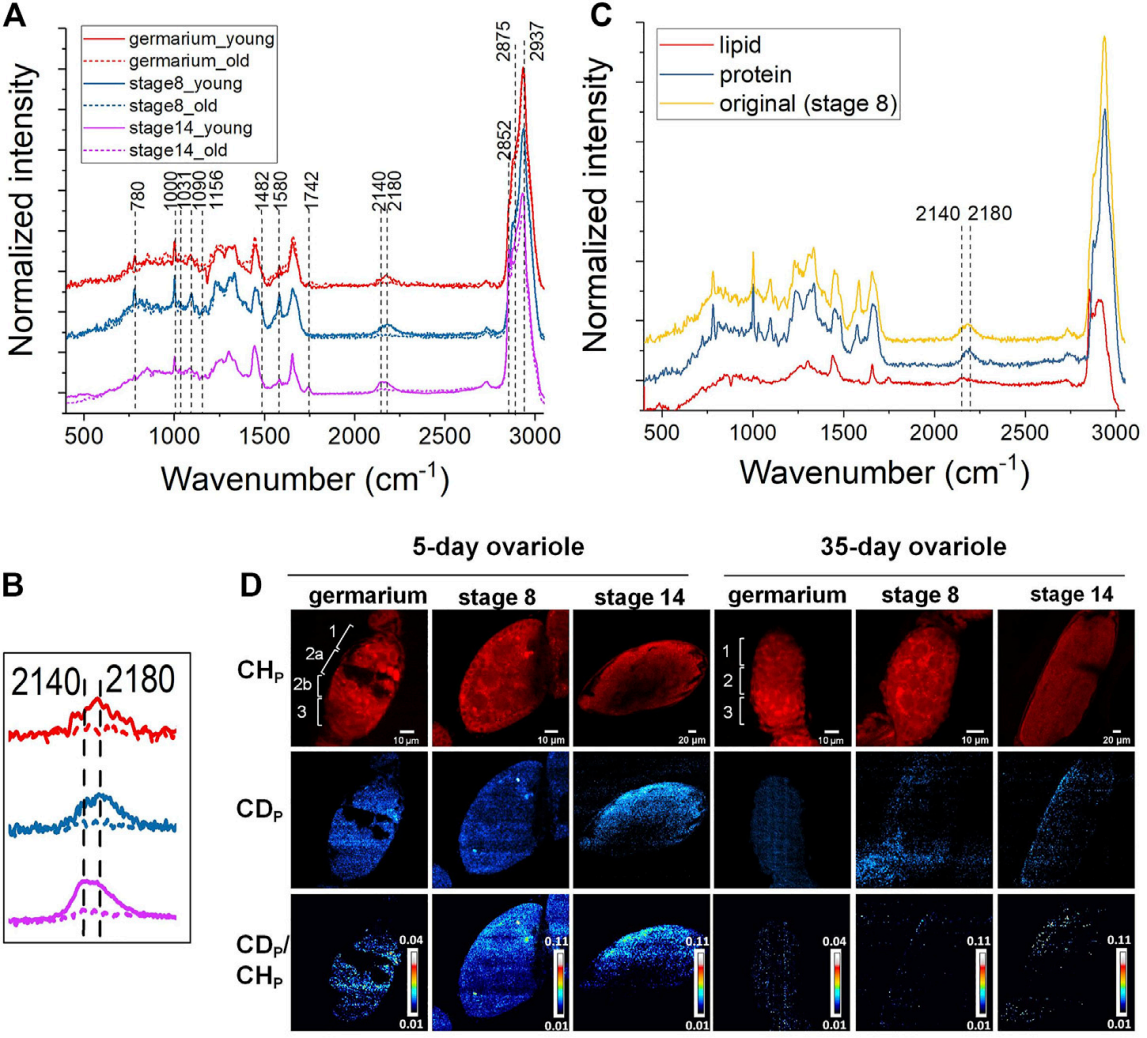
DO-Raman spectra and DO-SRS imaging of changes in lipid and protein metabolic activities in aging *Drosophila* ovary. **(A)** Averaged spectra of follicles at different developmental stages in 5- (solid line) and 35-day (dashed line) old flies (*n* = 10). The flies were treated with 20% D2O for 5 days. A decent CD signal was shown in the cell silent region. The CD intensity was greatly reduced in 35-day-old flies. **(B)** The C-D bond peaked at lower wavenumbers (∼2,140 cm^−1^) resembled newly synthesized lipids at stage 14, while the C-D peak acquired from the early developing follicles toward a higher wavenumber (around 2,180 cm^−1^) was mainly assigned to the newly synthesized proteins. Both CD proteins and lipids were reduced in the 35-day-old flies (dashed lines). **(C)** Normalized Raman spectra of follicles before (yellow) and after being treated with protease K (red) or washed with methanol (blue), respectively. Compared with the CD peak shown in the untreated spectrum (yellow), CD protein (blue) and lipid (red) peak positions were confirmed by dissolving lipids with methanol that abolished the peak at 2,140 cm^−1^ and removing proteins by proteinase K treatment that eliminated the peak at 2,180 cm^−1^, respectively. **(D)** DO-SRS imaging of protein metabolic dynamic in young and old *Drosophila* ovariole. DO-SRS images at 2,180 cm^−1^ displayed the subcellular localization of CD (newly synthesized) protein. In the same region of interest, SRS images at 2,937 cm^−1^ demonstrated the CH (total) proteins. The ratiometric images of CD_p_/CH_p_ were generated to show the location of newly synthesized proteins. The scale bars in germarium and stage 8 are 10 μm, and in stage 14 is 20 µm.

To address this, we applied DO-SRS imaging to visualize newly synthesized lipids and proteins and quantify their turnover rates in aging ovaries. Flies (0-day and 30-day after eclosion) were treated with 20% D_2_O for 5 days to obtain a detectable incorporation of CD signal for Raman imaging. After a 5-day labeling, decent CD signals were detected at each developmental stage (germarium, stage 1, stage 3, stage 5, stage 8, and stage 14) of the ovarioles in 5-day-old females. We acquired spectra from the fingerprint region (Figure 2A, Supplementary Figure S1A) and C–H region (Figure 2A, Supplementary Figure S1B), and observed proteins and lipids were dominated in developing follicles and mature oocytes, respectively, which were consistent with label-free Raman spectra that we collected previously. The C-D bond peak at 2,140 cm−^1^ resembled the newly synthesized lipids at stage 14, while the C-D peak acquired from the early developing follicles toward a higher wavenumber (around 2,180 cm^−1^) was mainly from the newly synthesized proteins (Figures 2A,B). The peak positions of CD proteins/lipids are consistent with that in fat body tissues and other biological systems as reported previously (Li et al., 2021; Singh and Keiderling, 1981). Furthermore, we reconfirmed these results by dissolving lipids using methanol that abolished the peak at 2,140 cm^−1^, and removing proteins by proteinase K treatment that eliminated the peak at 2,180 cm^−1^ (Figure 2C), respectively, which were consistent with our previous observations in mice (Shi et al., 2018).

Compared with the 5-day-old females, the CD signals from 35-day-old flies were largely reduced (Figures 2A,B, Supplementary Figure S2). This indicates that the amount of newly synthesized lipids (Supplementary Figures S2A, B, C) and proteins (Supplementary Figures S2D, E, F) was dramatically deceased in old ovaries. In the same region of interest, the SRS images of CH lipids and CH proteins were acquired at 2,852 cm^−1^ and 2,937 cm^−1^, respectively (Figure 2D, Supplementary Figure S1C).

For quantification, CD/CH was used as a ratiometric indicator for the amount of newly synthesized macromolecules normalized against variations among individuals and heterogeneity within the same tissue. From the results displayed in Figure 2D, Supplementary Figure S1C, we first visualized protein and lipid synthesis in *Drosophila* ovariole simultaneously during germline development and resolved their different metabolic dynamics. Previous studies reported that there was a large amount of lipids deposited inside the mature oocytes (Dunning et al., 2014; Ishigaki et al., 2017), however, it is unclear whether there is any difference between protein and lipid accumulation among oocytes and other earlier developing stages. In addition, there is no study on the metabolic activity changes of ovary during *Drosophila* aging. To better describe the distribution of different cell types in the germarium, it is divided into 4 discrete regions (regions 1, 2a, 2b, and 3) based on the overall morphology shown in the protein channel (2,937 cm^−1^) and according to the developmental stage of the nascent germline cyst and its derived oocyte reported in the literatures. Region 1 of the germarium contains the germ stem cell (GSC) niche and germline cysts of 2, 4, or 8 cells. Region 2a contains 16 cell germline cysts in which two pro-oocytes are determined. In region 2b, the nurse cells and oocyte are specified. As the cyst enters into region 3, the nurse cells are enlarged and the oocyte adheres to the posterior, and they are wrapped by surrounding follicle cells.

We found that 5-day-old flies showed active protein and lipid syntheses inside the ovariole at all the stages (Figure 2D, Supplementary Figure S1C). Earlier developing stages showed higher levels of protein synthesis, while no obvious difference of turnover rates among different cell types was observed. Our results demonstrated that in the young ovary where protein and lipid synthesis continuously increased throughout the germline development and reached the highest level in mature oocytes. Lipid and protein metabolism also showed different age-related dynamics in *Drosophila* ovary, consistent with the Raman spectra. The overall protein and lipid synthesis rate reduced dramatically in 35-day-old flies as compared with 5-day-old ones, indicating a decline in metabolic activity during aging.

### SRS Imaging of Lipid Subtype Distribution in Aging *Drosophila* Ovariole

The subcellular resolution and the molecular specificity of SRS microscopy also enabled us to differentiate distinct spatial patterns of different lipid subtypes. Previous studies reported that the 2,875 cm^−1^/2,852 cm^−1^ ratio can be used as an indicator for both conformational state and lateral packing of lipids (Choe et al., 2016; Uematsu and Shimizu, 2021). High values of this ratio have been associated with a compact organization and a solid phase of lipids, which is usually related to high content of saturated lipids (Matthews et al., 2010; Uematsu and Shimizu, 2021). The ratio of 3,005 cm^−1^/2,852 cm^−1^ corresponds to the relative level of unsaturated lipid to total lipids in the tissue (Toledo et al., 2016). To understand how lipid subtypes change during ovarian aging at the subcellular level, we applied label-free SRS imaging at 2,875 cm^−1^, 3,005 cm^−1^, and 2,852 cm^−1^, and generated ratiometric images to map the distribution of saturated and unsaturated lipids in three representative stages, the germarium, stage 8, and stage 14.

**FIGURE 3:**
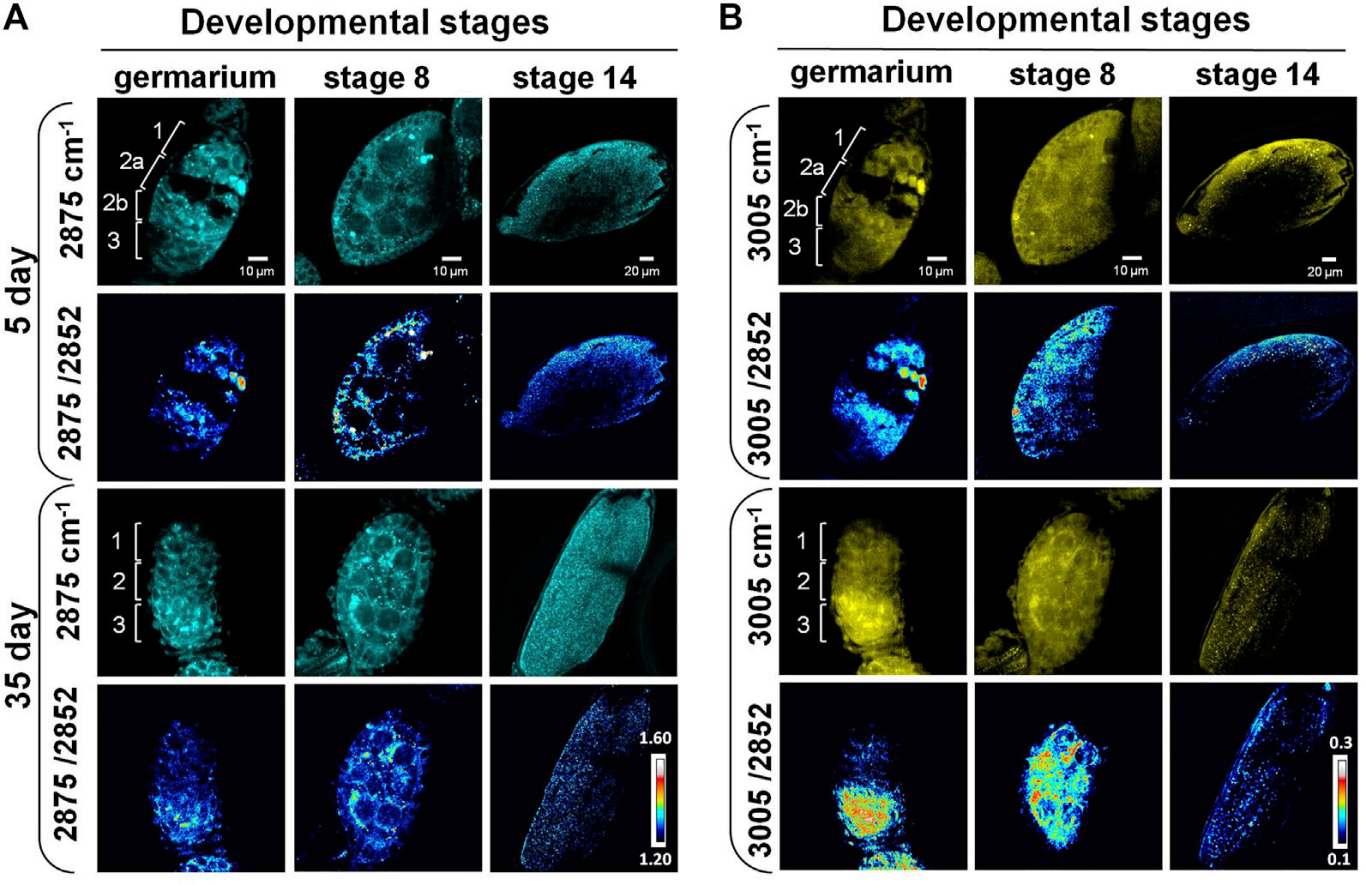
SRS imaging of lipid subtypes’ change in developing follicles of aging *Drosophila*. **(A)** SRS imaging at 2,875 cm^−1^ of 5- and 35-day ovaries. The ratiometric images of 2,875 cm^−1^/2,852 cm^−1^ were used to evaluate the concentration and distribution of saturated lipids. The images showed higher saturated lipid content in regions 1 and 2a of the germariums in young flies, but it was reduced in the germariums of 35-day-old flies. Saturated lipids in region 2b were mainly localized in the lipid droplets inside the nurse cells of 5-day-old flies, but they were condensed to the bar-shaped structures in 35-day-old flies. At stage 8, saturated lipid was visualized in the cytoplasm of germ cells in both young and old files, but it was reduced in the surrounding follicle cells in old files. The level of saturated lipids at stage 14 was reduced in old flies compared with young ones. Scale bar, 10 µm in germarium and stage 8, and 20 µm stage 14. **(B)** SRS imaging at 3,005 cm^−1^ of 5- and 35-day ovaries. The ratiometric images of 3,005 cm^−1^/2,852 cm^−1^ were used to evaluate the concentration and distribution of unsaturated lipids. Unsaturated lipids were largely reduced in region 1 of the germarium during fly aging. However, they tended to be accumulated in region 3 in old flies, where germ cells were localized. The distribution of unsaturated lipids is consistent in repeated experiments for stage 8 follicles in old flies. Compared with the young flies, the content of unsaturated lipids in old files germ cells (nurse cells and oocytes) was increased, but it was reduced in the surrounding follicle cells (somatic cells). The level of unsaturated lipids at stage 14 was reduced in old flies compared with the young ones. Scale bar, 10 µm in germarium and stage 8, and 20 µm stage 14.

As shown in Figure 3A, the lipids were mainly localized at regions 1, 2b, and 3 in the germarium of 5-day ovary. A cleft was clearly observed from the SRS images between regions 1 and 2b, where region 2a containing developing cysts was localized. Almost no SRS signals could be detected inside the cleft except some membrane structures, indicating that the macromolecular constitutions are different among these regions. However, the cleft disappeared in 35-day-old fly ovary, suggesting that chemical or structural changes happened in old females. Our SRS ratiometric images showed higher saturated lipid content in regions 1 and 2a of the germariums from young females, but the lipid content was largely reduced in the germariums of 35-day-old flies. Saturated lipids in region 2b were mainly localized in the lipid droplets inside the nurse cells from 5-day-old flies, which were condensed to the bar-shaped structures in the 35-day-old flies. The bar-shaped structure looks like an intercellular bridge between the adjacent cells, reminiscent of fusome that is localized in the developing cells of germline cysts. The fusome is an organelle conserved from invertebrates to mammals, which plays an important role in the synchronization of the cell cycle and controlling of the gamete quality by sharing substances between cells (Deng and Lin, 1997). Previous studies revealed that the fusome originated from endoplasmic reticulum related membrane vesicles and is associated with many proteins and organelles (Deng and Lin, 1997; Hsu et al., 2008; Hinnant et al., 2020). However, our images indicate an abundant amount of saturated lipids in this bar-shaped structure. Further study is necessary to determine this structure.

Similar to saturated lipids, the unsaturated lipids were largely reduced in region 1 of the germarium (Figure 3B), indicating that the total lipids were dramatically decreased in germ stem cell niche during aging. However, unsaturated lipids tend to be accumulated in region 3 of the old flies compared with young ones. Consistently, this phenomenon was also observed in stage 8 follicles of the old flies. In old flies, the amount of unsaturated lipids was increased in germ cells (nurse cells and oocytes) but was reduced in the surrounding follicle cells (somatic cells). This lipid distribution is different from that in the younger ones, which shows both types of lipids being localized in the outer-layer follicle cells (somatic cells) as well as inside the germ cells (germ cells). As the nutrition trafficking between follicle cells, nurse cells, and oocytes maintains the lipid homeostasis between the somatic and germ cells (Shkolnik et al., 2011; Zhang et al., 2019a; Zhang et al., 2019b), the decreased lipid level in follicle cells may indicate the disruption of molecular trafficking between these cells, leading to reduced number and quality of eggs.

Altogether, our label-free SRS images showed that the developing follicles in the 35-day-old flies had lower lipid contents and disrupted lipid distributions compared with the 5-day-old files. Other studies suggest that the development of oocyte depends on the nutritional support from nurse and somatic cells in the earlier stages (Bogliolo et al., 2013; Bertevello et al., 2020). Our Raman spectral data also suggest the molecular remodeling toward lipid accumulation happening in the last stages of oogenesis. Combined with the changes in lipid hemostasis observed from SRS imaging, we hypothesized that lipid metabolism also changed in mature eggs.

This is supported by our ratiometric images that the number of lipid (both saturated and unsaturated lipids) enriched particles in eggs from 5-day-old ovaries was significantly reduced in 35-day-old flies. The morphology of eggs from these two age groups also showed differences as well. The eggs generated from 5-day-old females were plump, whereas the 35-day-old females appeared slimmer, suggesting the decrease in biomass in the eggs from old females.

### SRS Imaging of Cytochrome c Distribution in Aging *Drosophila* Ovary

**FIGURE 4:**
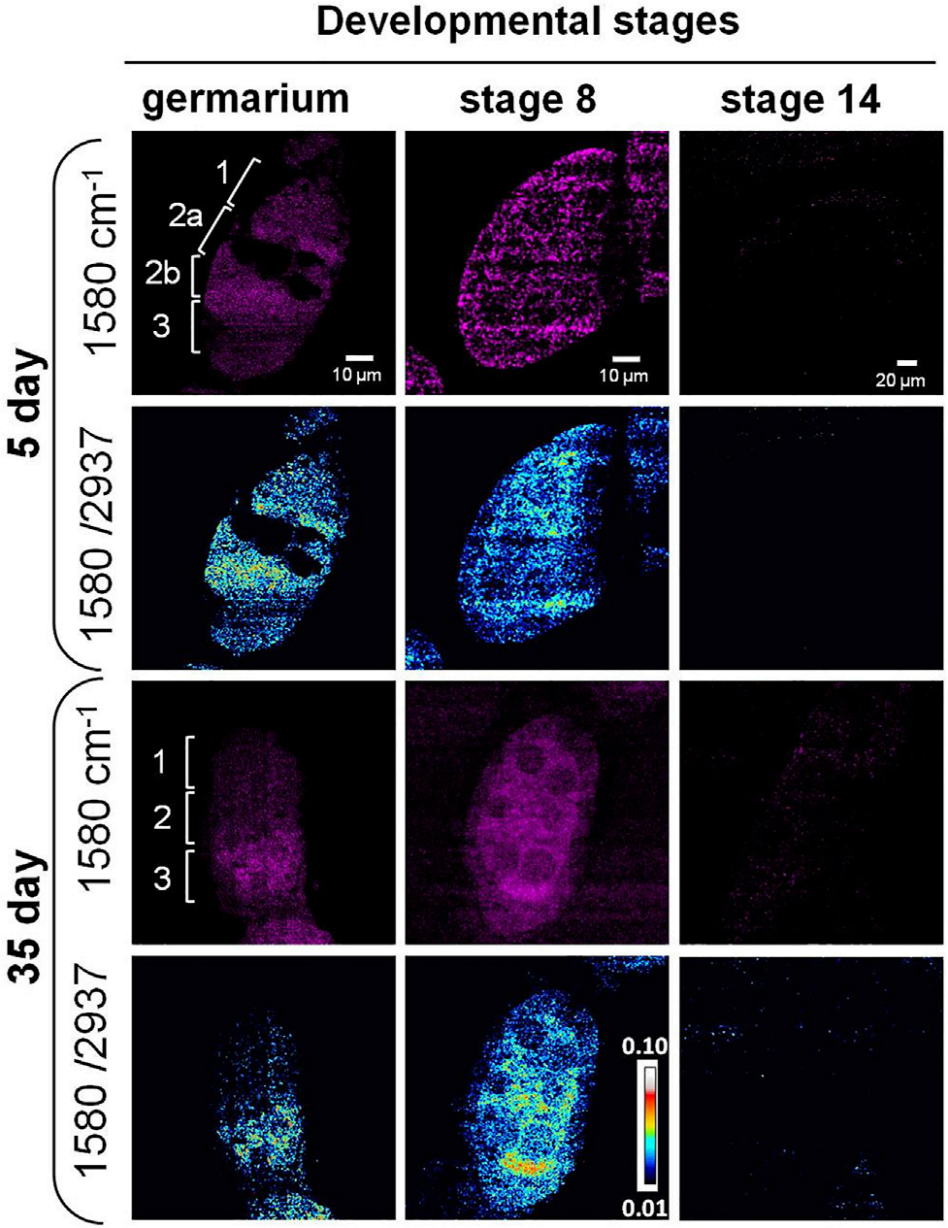
SRS imaging of Cytochrome c distribution in aging *Drosophila* ovary. SRS imaging at 1,580 cm^−1^ in 5- and 35-day ovaries. The ratiometric images of 1,580 cm^−1^/2,937 cm^−1^ were used to evaluate the concentration and distribution of Cytochrome c. In day 5 germarium, Cytochrome c was detected in the germ stem cell niche and the developing cysts. However, in old flies, the overall level of Cytochrome c was decreased, especially in region 1 where germ stem cell niche was localized. At stage 8, Cytochrome c showed a clear mitochondrial pattern in the nurse cells in both young and old ovaries, which is consistent with its *in situ* localization to the intermembrane space of these organelles. In the young ovary, Cytochrome c was evenly distributed in the cytoplasm of nurse and follicle cells at stage 8, while its signal level was upregulated in the nurse cells and reduced in the follicle cells in old ovaries. Inside the nurse cells, Cytochrome c was more evenly distributed in young flies but more condensed in old flies. Scale bar, 10 µm in germarium and stage 8, and 20 µm stage 14.

As previously mentioned, the Raman peak at 1,580 cm^−1^ was assigned to Cytochrome c or carotenoids. To identify the molecule, we treated the ovary with methanol that removed carotenoids. The peak still existed after the treatment (Figure 2C), indicating it was from the porphyrin ring of Cytochrome c (Kumamoto et al., 2018; Hniopek et al., 2021). Using SRS microscopy, we next imaged Cytochrome c in developing germ cells to probe its change during aging. Among the stages that we observed, the signal from 1,580 cm^−1^ mainly appeared in the germarium and stage 8, but not at stage 14 in both young and old flies (Figure 4). This is consistent with the Raman spectra we measured where the 1,580 cm^−1^ peak existed only in the developing follicles but not in the mature oocytes. To semi-quantitatively analyze subcellular level of Cytochrome c between young and old flies, we normalized the intensity of 1,580 cm^−1^ to the whole protein level at 2,937 cm^−1^. As shown in Figure 4, in 5-day young flies germarium, Cytochrome c exist in germ stem cell niche and the developing cysts, while in the old flies, the total level of Cytochrome c was decreased, especially in region 1 where germ stem cell niche was localized. At stage 8, Cytochrome c showed a clear mitochondrial pattern in the nurse cells in both young and old ovaries, which is consistent with its *in situ* localization to the intermembrane space of these organelles. In the young ovary, Cytochrome c was evenly distributed in the cytoplasm of nurse and follicle cells. Interestingly, Cytochrome c signal was upregulated in the nurse cells and reduced in the follicle cells in old ovaries.

Apart from the signal level changes of Cytochrome c, the signal distribution also showed differences between young and old flies. Cytochrome c was more evenly distributed in young flies but more condensed in old flies, suggesting the change in mitochondrial structure with aging. It has been reported that the fusion and fission dynamics of mitochondrial are changed during aging in *Drosophila* ovary (Ishihara et al., 2009; Sênos Demarco et al., 2019; Sênos Demarco and Jones, 2019), and altered mitochondrial biogenesis happens during oogenesis in other model systems (Dunning et al., 2014; Garcez et al., 2020). The change in Cytochrome c during aging may lead to altered mitochondrial dynamics (Varkey et al., 1999). Our results suggest that SRS imaging can be used as a powerful tool to monitor mitochondrial dynamics and function *in situ*.

Cytochrome c release is an important event in the intrinsic pathway of apoptosis. In the late-stage egg chambers, nurse cells transfer their cytoplasmic contents to the developing oocytes and undergo apoptotic cell death. Cytochrome c is released from the mitochondria and can result in the activation of caspases (Martin and Baehrecke, 2004; Pritchett et al., 2009). Active caspases can be detected in region 2 of the germarium using an anti-active caspase-3 antibody, and mutants lacking the effector caspase show a reduced level of DNA fragmentation and autophagy in region 2 when compared with the wild type. Studies also showed high levels of caspase-mediated cell death in mid-oogenesis (Peterson et al., 2003; Mazzalupo and Cooley, 2006). Further functional studies are needed to elucidate the relationship between aging-dependent Cytochrome c change and ovary function.

## Discussion

The development of the *Drosophila* oocytes is a very complex process where it begins from a syncytium that is formed by 16 sister cells derived from four rounds of incomplete division of a single germ cell. These 16 cells share the same cytoplasm. Only one of them will become the oocyte, while the remaining 15 cells differentiate to nurse cells, which fuel the oocyte with nutrients and cytoplasmic components (Doherty et al., 2021). This means that the polarization of the cyst cytoplasm and membrane are involved in the selection of the oocyte (Theurkauf, 1994). The polarization of the oocyte is intimately associated with the establishment of the body axes of the fly and the following embryo development (Huynh and St Johnston, 2004). All these processes depend on reciprocal interactions between germ cells and their somatic niche (Bertevello et al., 2020). Many factors and signaling pathways are involved in the processes of oocyte determination (Zimon et al., 2006; Perrimon et al., 2012). One of them is Notch signaling pathway that plays an important role in controlling cell proliferation and differentiation through cell-cell interaction (Zimon et al., 2006). However, whether and how lipid metabolism affects the cell determination remains unclear. Our label-free SRS imaging results show that different types of cells have diverse lipid profiles in the germ stem cell niche as well as the developing follicles, and the level and distribution of these lipid components are changed during development and aging (Figures 3A,B).

During *Drosophila* aging the division of stem cells reduces dramatically, which is coincident with decreased egg production and fly infertility (Zimon et al., 2006; Shi et al., 2016; Dipali et al., 2019). However, it is still unclear if this reproductive defect is cell autonomous or nonautonomous. Apoptosis and resorption of developing egg chambers could also lead to the decrease in fertility (Drummond-Barbosa and Spradling, 2001). If the early aging stages of these events could be measured, it would provide valuable information to prevent or slow down the decline processes of the ovary. Methods have been developed to genetically study the survival of stem cells, division rate, and function during aging in adult *Drosophila*. Studies indicated cellular stresses such as oxidative and heat shock, endocrine and insulin signaling, epigenetic modification and chromatin structure, and metabolic homeostasis as major factors involved in the reproduction maintenances (Hsu et al., 2008; Bogliolo et al., 2013; Mok et al., 2016; Shi et al., 2016; Jeong et al., 2019; Sasaki et al., 2019). This indicates that the internal and external factors work together to determine the ovary aging processes. In our study, DO-SRS was used to sensitively monitor the metabolic activity change in developing follicles during aging (Figure 2, Supplementary Figure S1 and Supplementary Figure S2).

Cytochrome c, which is a protein important for mitochondrial function and commonly used as a mitochondrial marker (Schägger, 2002), could be detected by Raman and SRS microscopy. Previous electron microscopy (EM) (Garcez et al., 2020) and immunostaining fluorescence studies have documented that GSCs in region 1 of germarium have a predominantly small punctate mitochondria, and that mitochondria progressively becomes more aggregated in regions 2b and 3, consistent with an increase in mitochondria fusion (Zhang et al., 2019a; Zhang et al., 2019b; Sênos Demarco et al., 2019; Amartuvshin et al., 2020; Chen et al., 2003). This increase in mitochondria fusion along with cell differentiation suggests that mitochondrial dynamics may play a role in regulating cell fate. Another study showed that mitochondrial dynamics also changes during the aging process with an increase in fission and decrease in fusion in *Drosophila* (Amartuvshin et al., 2020). Our results consistently show that the aging ovarioles have a condensed signal of Cytochrome c, which may be due to the dynamics change of the mitochondria (Figure 4). We also detected that the level of Cytochrome c was reduced in the developing follicles in aged flies (Figure 4). As Cytochrome c is an essential component of the electron transport chain, reduction of Cytochrome c indicates that the mitochondrial function may be compromised during aging in the ovary. Our results show that unsaturated lipids and Cytochrome c were accumulated simultaneously in egg chambers from old flies, especially in stage 8 (Figures 3B, 4). Previous studies demonstrated some types of lipids could induce cell apoptosis or non-apoptosis-dependent cell death (Knoblich, 2008; Pritchett et al., 2009; Park et al., 2018; Liang et al., 2020). Further studies are necessary to investigate if the interplay between unsaturated lipids and Cytochrome c is involved in aging-dependent malfunction of ovary.

For the first time, using label-free and D_2_O-probed Raman and SRS imaging we revealed lipid metabolism in the ovary during *Drosophila* aging (Figures 1B–E and Figure 2A). For future studies, it will be interesting to understand how these metabolic changes are related to the reproductive decline in *Drosophila*, the causes of such decline, and whether these changes will be conserved among different species. To address these questions, genetic or diet manipulation can be potentially used to identify new genes or food components that can modulate the reproductive life span. Genetic studies have begun to uncover genes regulating reproductive life span (Rose and Charlesworth, 1981; Helfand and Rogina, 2003; Partridge et al., 2005). The high glucose and protein-enriched foods were reported to change (May et al., 2015; Zanco et al., 2021) the reproductive status of *Drosophila*, even though the underlying mechanisms are still unclear. DO-Raman/SRS demonstrates a broad application in monitoring metabolic changes in tissues and cell *in situ*, toward unraveling the underlying mechanisms between metabolism, reproduction, and aging.

## Materials and Methods

### Fly Stocks

Wild type (*w* ^*1118*^ stock #5905) were originally obtained from the Bloomington Stock Center and have been maintained in the lab for several generations.

### D_2_O-Labeling Experiments

The metabolic activity changes of wild-type flies at different ages were labeled by transferring the 0-day, and 30-day (after eclosion) female adult flies to the 20% D_2_O labeled corresponding food conditions for 5 days, then the 5-day, and 35-day aged flies were sacrificed, and ovaries were dissected and subjected to Raman measurements and SRS imaging.

### *Drosophila* Sample Preparation for Raman and SRS Microscopy

Ovary tissues were dissected in PBS and fixed in 4% formaldehyde for 30 min at room temperature (RT). After fixation, tissues were washed four times with PBS in glass wells. Tissues were then sandwiched between a cover slide and bottom slide with PBS solution. To prevent the tissue drying, nail polish was used to seal the surrounding of cover slides.

### Spontaneous Raman Spectroscopy

Raman spectra of all the tissue samples were measured by a Raman spectrometer connected to a confocal Raman microscope (XploRA PLUS, Horiba). A 532 nm diode line focus laser (∼40 mW at the sample) was focused on the cells with the help of a ×100 objective (MPLN×100, Olympus). The laser power on the sample was optimized to avoid any damage to the cells. A cooled charge coupled device (CCD) detector fitted to a 2,400 grooves/mm grating spectrometer was used to detect the signal. Spectra were collected at 60 s acquisitions with an accumulation of 2. The background spectra were taken for each tissue point at the same focus plane and were subtracted from original spectrum immediately. All ratio calculations were carried out on the raw data before any normalization or baseline correction steps. Peaks were normalized to the phenylalanine peak at 1,003 cm^−1^. The instrumental calibration was verified using the silicon line at 520 cm^−1^. The observed data were processed and analyzed using Prism software (Origin Lab Corporation, Northampton, MA).

### Stimulated Raman Scattering Microscopy

An upright laser-scanning microscope (DIY multiphoton, Olympus) with a ×25 water objective (XLPLN, WMP2, 1.05 NA, Olympus) was applied for near-IR throughput. Synchronized pulsed pump beam (tunable 720–990 nm wavelength, 5–6 ps pulse width, and 80 MHz repetition rate) and Stokes (wavelength at 1032 nm, 6 ps pulse width, and 80 MHz repetition rate) were supplied by a picoEmerald system (Applied Physics and Electronics) and coupled into the microscope. The pump and Stokes beams were collected in transmission by a high NA oil condenser (1.4 NA). A high O. D. shortpass filter (950 nm, Thorlabs) was used that would completely block the Stokes beam and transmit the pump beam only onto a Si photodiode for detecting the stimulated Raman loss signal. The output current from the photodiode was terminated, filtered, and demodulated by a lock-in amplifier at 20 MHz. The demodulated signal was fed into the FV3000 software module FV-OSR (Olympus) to form image during laser scanning. All images obtained were 512 × 512 pixels, with a dwell time of 80 μs and an imaging speed of ∼23 s per image. A background image was acquired at 1900 cm^−1^ and subtracted from all SRS images using Fiji (ImageJ).

### Statistical Analysis

Student *t*-test was performed for statistical analysis using GraphPad Prism software. Ratio data were analyzed from raw intensity values without baseline correction and normalization.

## Data Availability

The original contributions presented in the study are included in the article/Supplementary Material, further inquiries can be directed to the corresponding author.

## Supplementary Material

Supplementary Figure S1 DO-Raman and DO-SRS imaging of lipid metabolic activity changes in aging *Drosophila* ovary. The fingerprint region **(A)** and CH stretching region **(B)** of the Raman spectra of follicles at different developmental stages in 5- and 35-day flies. were enlarged to show the molecular bond difference between young and old flies. **(C)** DO-SRS imaging of CD lipids (newly-synthesized) at 2140 cm^−1^. In the same region of interest, SRS images of CH lipids (total lipids) were acquired at 2852 cm^-1^. The ratiometric images of CD_L_/CH_L_ were generated to show the location of newly-synthesized lipids. Scale bar, 10 µm in germarium and stage 8, and 20 µm stage 14.

Supplementary Figure S2 Quantification of lipid and protein turnover rates in Drosophila ovary. At all stages, both lipid and protein turnover rates were significantly reduced in aging *Drosophila.* Raman spectra of germarium, stage 8 and stage 14 were collected from 5-day (*n* = 5) and 35-day (*n* = 5) Drosophila, respectively. Lipid **(A,B,C)** and protein **(D,E,F)** turnover rates were calculated and analyzed to compare the statistical significance between young and old flies. Results shown were mean ± SD. ****, *p* < 0.0001 using unpaired *t*-test.
