# Supplementary figures and images for: Direct Imaging of Lipid Metabolic Changes in Drosophila Ovary During Aging Using DO-SRS Microscopy
Source: Front Aging. 2022 Feb 3;2:819903. doi: 10.3389/fragi.2021.819903 (PMC9261447; doi:10.3389/fragi.2021.819903)

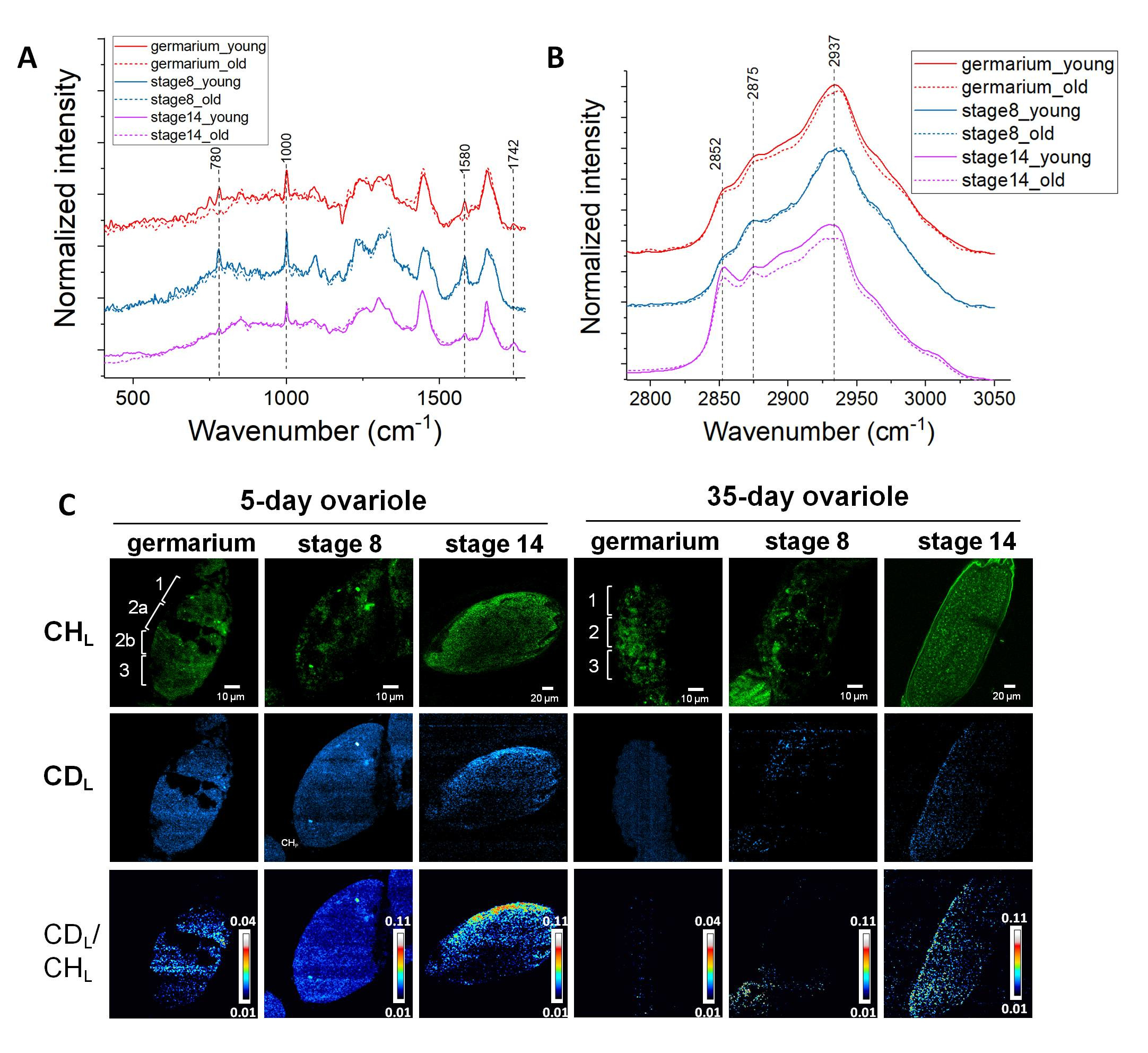

Supplement: Supplementary file 1 [file Image1.JPEG]

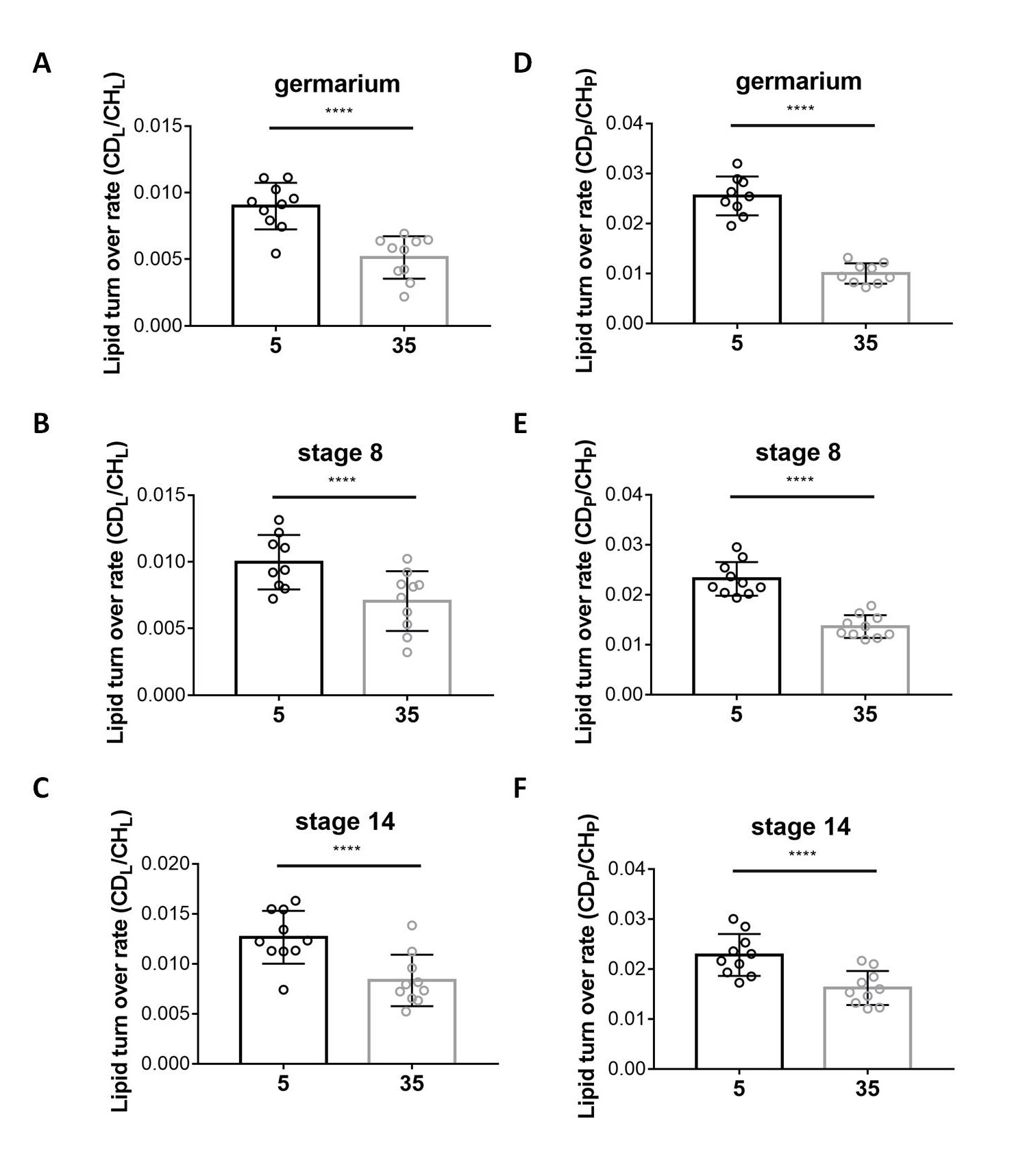

Supplement: Supplementary file 2 [file Image2.JPEG]
